# An Integrated Photogrammetric and Spatial Database Management System for Producing Fully Structured Data Using Aerial and Remote Sensing Images

**DOI:** 10.3390/s90402320

**Published:** 2009-03-30

**Authors:** Farshid Farnood Ahmadi, Hamid Ebadi

**Affiliations:** K.N. Toosi University of Technology, Faculty of Geodesy and Geomatics Engineering, Department of Photogrammetry and Remote Sensing, Tehran, Iran; E-Mail: ebadi@kntu.ac.ir

**Keywords:** DBMS, GIS, Integration, Photogrammetry, Remote Sensing, Spatial database, Topological relations

## Abstract

3D spatial data acquired from aerial and remote sensing images by photogrammetric techniques is one of the most accurate and economic data sources for GIS, map production, and spatial data updating. However, there are still many problems concerning storage, structuring and appropriate management of spatial data obtained using these techniques. According to the capabilities of spatial database management systems (SDBMSs); direct integration of photogrammetric and spatial database management systems can save time and cost of producing and updating digital maps. This integration is accomplished by replacing digital maps with a single spatial database. Applying spatial databases overcomes the problem of managing spatial and attributes data in a coupled approach. This management approach is one of the main problems in GISs for using map products of photogrammetric workstations. Also by the means of these integrated systems, providing structured spatial data, based on OGC (Open GIS Consortium) standards and topological relations between different feature classes, is possible at the time of feature digitizing process. In this paper, the integration of photogrammetric systems and SDBMSs is evaluated. Then, different levels of integration are described. Finally design, implementation and test of a software package called Integrated Photogrammetric and Oracle Spatial Systems (IPOSS) is presented.

## 1. Introduction

3D spatial data acquired from aerial and remote sensing images by applying photogrammetric techniques is one of the most accurate and economic data sources for GIS, map production and spatial data updating. Nowadays, the importance of imagery as a resource for obtaining spatial data is increasing, so that in the near future, around 50% of the existing data in organizations related to management of spatial information will be obtained by this approach [8].

Irrespective of the importance of photogrammetric techniques for spatial data production, there are many problems concerning storage, structuring, and appropriate management of spatial data obtained using the techniques. Some of these problems are:

- Cost of storing, retrieving and updating spatial data
- Data redundancy and inconsistency
- Existence of different types of errors on produced data
- Dependency between spatial data storage methods and applications
- Inappropriate access to available data
- Difficulty in storing and managing spatial and attribute data in a seamless manner
- Difficulty in adding attribute data to spatial data at the time of spatial data production

Spatial database management systems (SDBMSs) are able to store and manage large amounts of spatial and attribute data in an integrated form. They are also capable to reduce some of above mentioned problems. Therefore, direct integration of photogrammetric and spatial database management systems may save time and reduce cost of producing and updating digital maps.

In the next section, necessity and importance of photogrammetric systems and SDBMSs integration will be discussed. Section 3 explains integration methods of the systems. In Section 4, the new method for direct integration of the systems is presented. Based on this method, design, implementation, and test of a software package called Integrated Photogrammetric and Oracle Spatial Systems (IPOSS) is discussed. Finally Section 5 includes the conclusions and some recommendations for future studies.

## 2. Necessity and Importance of SDBMS and Photogrammetric Systems Integration

### 2.1. Central Database Instead of Digital Maps

Nowadays, the methods used by most organizations for producing spatial data using photogrammetric techniques store data as a digital map. These digital maps are produced at special scales and for defined applications. Therefore, features displayed in these maps, are selected based on its specific application and do not include all features in the field. In this case, spatial data must be collected again for producing maps with different applications, or generalization operations should be carried out for generating maps at smaller scales. Both of these steps are costly and time consuming.

The expensive nature of spatial data production at special scales and for defined applications encourages development of new directions to use a central database for preparing, storing and updating a georeferenced model of the World [1]. By using a method which stores acquired data from photogrammetric systems, directly in a spatial database, different maps can be produced using stored data in the database. Thus, time and cost can be saved in the map production and updating stages of a project.

### 2.2. Preparing Data for On-line GIS

Some of the requirements which lead to online GIS development include:

- Data sharing and reusing of available spatial information
- Access to different data sources for choosing the most appropriate data for desired applications
- Applying some basic spatial analysis in the World Wide Web

In an on-line GIS, spatial data is stored in a central database on the basis of defined standards for data exchange via the web [7]. Therefore, photogrammetric systems, used for producing spatial data for GIS, should be able to store spatial data according to the defined standards simultaneously during the feature digitizing process.

### 2.3. Possibility of Removing Spatial Errors during Digitizing Process

Error removal and production of fully structured data during feature digitizing from aerial and remote sensing images eliminate the spatial data editing process. Thus, spatial data can be used for modeling or GIS analyses with minimal editing [3].

Structuring and semantic errors are two kinds of frequent spatial errors in spatial information systems. Structuring errors depend on the data model and the data structure of spatial information systems, so no unique method can be used to remove structuring errors for all systems. Some structuring errors, such as non polygon closure, can be detected and corrected using geometric or logical constraints [3].

Semantic errors are initiated from the real world description and can not be found without using the semantics of the real world entities. Topological errors are the most common types of semantic errors. These errors are caused by definition of topological relations between features that are inconsistent with the real world. Topological relations are of great importance in GIS [2–4]. Errors contained in GIS usually come from erroneous topological relations among spatial objects [9]. Definition of constraints to illustrate forbidden or unacceptable topological relations can be used to detect topological errors. By integration of photogrammetric systems and spatial databases, and applying capabilities of SDBMSs for spatial constraints evolution, some structuring errors and most semantic errors are prevented.

### 2.4. Improvement of Spatial Data Updating Process

Spatial data obtained using photogrammetric techniques from aerial and remote sensing images, is one of the most important sources for updating spatial databases. SDBMSs have capabilities to share data and generate different versions of stored data for updating process. Therefore, by integration of the systems, updating process for different parts of a region can be carried out simultaneously using several photogrammetric workstations. In this method, problems such as complexity of fusing different data and reduction of spatial data accuracy during editing procedure will be omitted [1].

### 2.5. Storing and Managing Spatial and Attribute Data as a Seamless Database

For those GIS packages which accept data in the form of digital map, a coupled approach is used to manage spatial and attribute data. In this approach, two systems are available [11]:

- RDBMS for managing attribute data
- Special module for managing spatial data

This approach is not efficient for the following reasons [11]:

- Inconsistency between two different models makes using and fusing of data difficult
- Some of DBMS services such as: retrieving, optimization and query techniques are unusable for spatial data.

Integration of photogrammetric systems and SDBMS is capable to store spatial and attribute data seamlessly and also causes to store spatial data directly in spatial databases. In this case, the problems arising from using coupled approach to manage spatial and attribute data are omitted.

### 2.6. Adding Attribute Data to Spatial Data at the Time of Spatial Data Production

3D models formed in photogrammetric workstations using aerial/remote sensing images can be used as an appropriate source for extracting some attributes. Therefore, integration of photogrammetric systems and SDBMSs enables to add simultaneously some attribute data to spatial data during the feature digitizing process.

## 3. Integration of Photogrammetric Systems and SDBMSs

Integration of photogrammetric systems and SDBMSs is a new approach and there is a few researches in literature on this field. Woodsford has evaluated integration of the systems and explained the necessity of a new method for direct integration of them [12]. Also Heipke has described integration of photogrammetric systems and SDBMSs as a requirement which an ideal GIS must meet to cope with the challenges of the future [8].

The method which already has been used in commercial systems is indirect integration (File Based Exchanging of Spatial Data). In this method, no direct relationship is available between the photogrammetric system and the SDBMS, and an integration process is carried out via file based exchanging of spatial data [12]. These files are outputs of photogrammetric systems in Computer Aided and Design (CAD) or GIS standard formats. In this method, updating of spatial data is carried out based on general replacement or using the file of removed and generated features. The most important problems of indirect integration method are [12]:

- Probability of missing some data during data reading and transferring process
- Time consumption for retrieval, evaluation and editing of spatial data

A method which can establish a direct connection between photogrammetric systems and SDBMSs, overcomes most problems of indirect integrated systems. The method discussed in Section 4 is a new method for direct integration of the systems. We have applied the method to design and implement a software package called IPOSS.

## 4. Direct Integration of Photogrammetric Systems and SDBMSs

In direct integration of photogrammetric systems and SDMBSs, a connecting interface is used to integrate photogrammetric systems and SDBMSs. Thus, generation of this interface is the first step for instantaneous integration of the systems. The interface system has a critical role in integration of the systems and its architecture determines the integrated system characteristics. This method solves most problems of the indirect integration method. For example; spatial data evaluation and structuring can be carried out according to the defined rules and constraints simultaneously during the data transferring.

In this research, the integrated system designed and implemented based on the new method includes three following parts:

- SDBMS
- Photogrammetric system
- Interface system

Choosing a proper SDBMS, necessity of using CAD environment as an interface for data receiving from photogrammetric systems and architecture of the integrated system will be explained in the following sections.

### 4.1. Oracle Spatial as a SDBMS

Oracle Spatial is a SDBMS which supports Standard Query Language (SQL) and a collection of special functions to facilitate storage, retrieving, manipulation and updating of spatial data. In Oracle Spatial, Open GIS Consortium (OGC) standards can be completely implemented for simple features. Also some of its abilities such as [10]:

- Making use of the 9-intersection model [5–6] in topological analysis
- Some tools for manipulation of spatial data in a large database
- Producing backup versions of spatial data very fast
- Retrieval techniques for spatial data on the web or internet are available for users of this system.

SQL of Oracle Spatial was developed in a way that spatial queries can be performed simply and efficiently [11]. Oracle Spatial is consistent with ESRI products and used widely in GIS. Data exchanges between these systems can also be performed easily [13]. Oracle objects can be embedded in the programming languages support COM and ActiveX standards. These programming languages can load existent data in Oracle database. Mentioned capabilities of Oracle Spatial make it as a very powerful SDBMS for integration with photogrammetric systems.

### 4.2. Using Computer Aided and Design (CAD) Environment as an Interface for Receiving Data

The first step for direct integration of photogrammetric systems and Oracle Spatial is to simultaneously transfer extracted data from the photogrammetric model to Oracle Spatial during the feature digitizing process. For this purpose, simultaneous control of both systems via an interface that can access and use operators of both systems is necessary.

A method for implementation of such an interface is to use programming languages with Object Link Embedding (OLE) capability. For using this capability, both systems should embed their objects in these programming languages. As mentioned in the previous section, this capability is supported in Oracle Spatial. Most photogrammetric systems do not deliver such capabilities for users. Also access to the source code of these systems for changing the format of produced data and the output environment is not possible.

**Figure 1.**
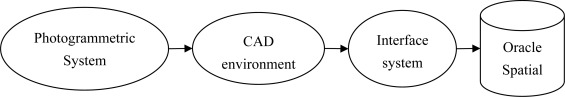
CAD environment as an interface for receiving data points sent from photogrammetric systems.

Nowadays, most photogrammetric systems use standard CAD environments as their digitizing environment. In these photogrammetric systems, data points extracted from photogrammetric models are sent to the CAD environment via a device called Stereo-link. Currently, most CAD environments such as AutoCAD and Microstation support programming languages with OLE capabilities. Thus, according to the limitations mentioned for the photogrammetric systems, CAD systems can be used as an interface to receive data points (Figure 1).

Most photogrammetric systems use Microstation as a standard CAD environment for the digitizing process [3]. Thus in this research, Microstation has been selected as an interface environment.

### 4.3. Integrated Photogrammetric and Oracle Spatial Systems (IPOSS)

For direct integration of Oracle Spatial with photogrammetric systems which use Microstation for the feature digitizing operation, the interface system should have the following characteristics and capabilities:

- Receiving 3D points extracted from the photogrammetric model simultaneously during feature digitizing process from aerial and remote sensing images.
- Producing structured data sets from received points according to OGC standards and topological relations between features in the real world
- Generating required tables for storing spatial and attribute data and retrieving them seamlessly according to the user requirement.
- Storing spatial and attribute data in a recognizable format by Oracle Spatial
- Accessing Oracle Spatial capabilities simultaneously during feature digitizing process

**Figure 2.**
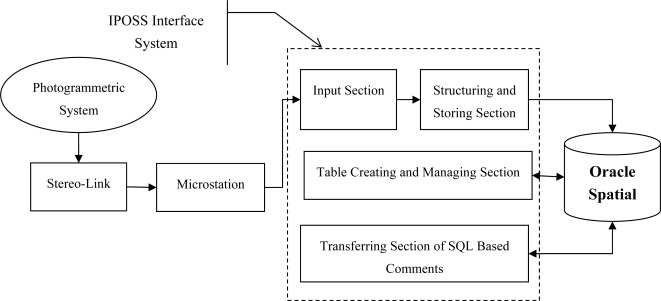
Main structure of the interface system

Accordingly, in this research four major sections including:

- Input Section
- Structuring and Storing Section
- Table Creating and Managing Section
- Transferring Section of SQL Based Comments

are considered in the general comprehensive scheme of IPOSS. Figure 2 illustrates the main structure of the interface system.

#### 4.3.1. Input Section

The input section of the interface system enables the system to receive data points which have been sent from the photogrammetric system. The received points are sent to the structuring and storing section simultaneously during feature digitizing process from aerial and remote sensing images.

**Figure 3.**
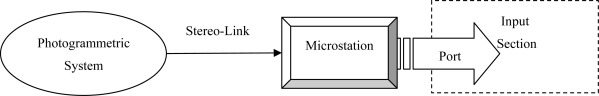
Main structure of IPOSS Input Section

The input section of the interface system is designed based on photogrammetric systems such as PhotoMod, which uses the Microstation for feature digitizing. PhotoMod supports both aerial and remote sensing images. For receiving and sending data points, a software port has been designed and implemented in the input section. Main structure of the input section is shown in Figure 3. Stereo-Link transfers photogrammetric 3D model to Microstation. The port which is active in Microstation gets data points during feature digitizing process and enters them to the input section.

#### 4.3.2. Structuring and Storing Section

This section is one of the basic parts of the interface system and prevents some structuring errors and most semantic errors simultaneously during feature digitizing process. Also in this section, spatial data is stored according to OGC standards and recognizable formats via Oracle Spatial. It consists of following two subsections:

- Error Removal
- Storing Structured Features

##### 4.3.2.1. Error Removal Subsection

**Figure 4.**
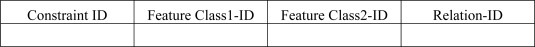
Structure of the table designed for storing defined constraints elements.

In this subsection, the detection of topological semantic errors is carried out by definition of forbidden or unacceptable topological relations between different classes of features. This subsection includes two following units:

- Unit of topological constraints definition: In this unit topological constraints required for topological error detection are defined by the user. The unit has been designed based on the 9-intersection model and enables users to define all forbidden or unaccepted topological relations between feature classes. The 9-intersection model has been implemented in Oracle Spatial as a powerful model for topological analysis. In this unit, a user interface has been considered for definition of the required constraints as following:
  Forbidden topological relation: feature class1, relation, feature class 2
  The relation between two feature classes can be defined as:
  ○ Disjoin
  ○ Touch
  ○ Overlap
  ○ Equal
  ○ Contains
  ○ Covers
  ○ Covered by
  ○ Inside
  ○ On
  The elements of defined constraints are stored in the table designed in Oracle database. The structure of this table is shown in Figure 4. Defined characteristics for the table columns help SDBMS to store defined constrains systematically, select proper constraints, reconstruct defined constrains using their elements and apply them for decision making process.
- Unit of error detection: By activation of a feature class as a selected feature for digitizing, a search process is carried out on the constraints table and feature classes related to the active feature class is extracted. By adding a new data point to the feature, topological relations between this feature and related feature classes are determined based on the 9-intersection model. In order to prevent error generation the latest added point is removed after a forbidden or unacceptable topological relation is detected. Then, an alert message is sent to the user and the interface system waits for a new data point.

**Figure 5.**
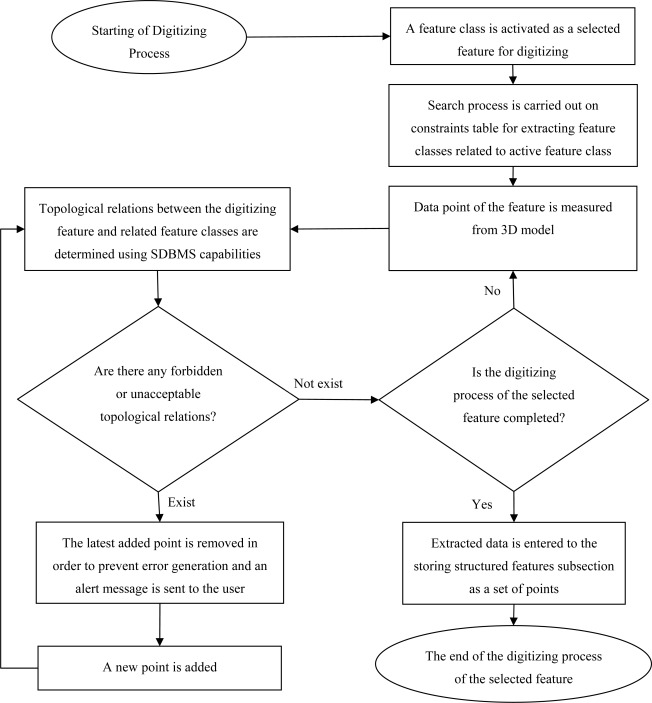
The workflow of error removal subsection

The workflow of error removal subsection is shown in Figure 5.

##### 4.3.2.2. Storing Structured Features Subsection

Spatial data sent from the error removal subsection is entered to the storing structured feature subsection as a set of points. This section structures the points set as a point, line or polygon feature according to the OGC standard and stores them in a recognizable format via Oracle Spatial.

#### 4.3.3. Table Creating and Managing Section

This section has a double-edge relationship with Oracle Spatial and enables users to create required tables for storing any group of features by the means of a connecting channel. The channel is created between the interface system and Oracle Spatial.

#### 4.3.4. Transferring Section of SQL Based Comments

This section has a double-edge relationship with Oracle Spatial. This relationship enables users to apply functions and operators of Oracle Spatial for spatial analysis, recognition of topological relations, spatial indexing, accuracy control and etc. by the use of SQL comments simultaneously during feature digitizing process.

### 4.4. Implementation of IPOSS

In this research, Visual Basic has been used for implementation of IPOSS. IPOSS establishes a connection between PhotoMod as a photogrammetric workstation and Oracle Spatial. After IPOSS activation, its user interface appears in the PhotoMod environment and user can apply it for:

- Creating and editing required tables for storing spatial and attribute data
- Definition of forbidden or unaccepted topological relations between feature classes
- Definition of required settings and parameters for feature digitizing and storing them in the database
- Spatial querying

**Figure 6.**
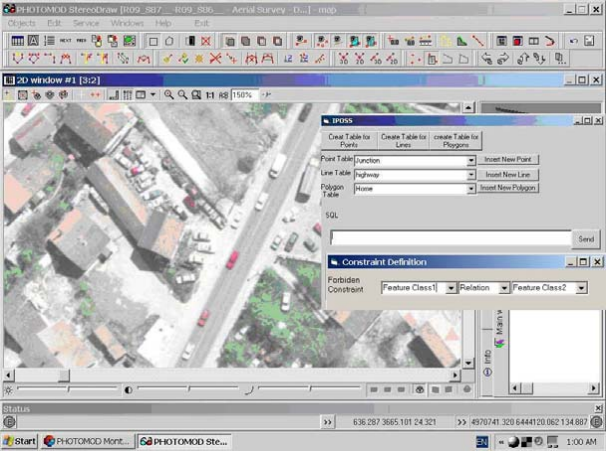
User interface of IPOSS in PhotoMod environment

Figure 6 shows user interface of IPOSS in PhotoMod environment.

### 4.5. IPOSS Test

IPOSS test is discussed in two parts. The following subsection presents the performed method for general test of the system for producing spatial data of different feature classes in an urban area. The next subsection illustrates the system operation during building feature digitizing process as an applied example.

#### 4.5.1. General Test of IPOSS

For a general test of IPOSS, a 3D model of an urban area was created in PhotoMod using aerial images at a scale of 1:8,000. Then the digitizing operation for each feature (point, line and polygon) in all available classes (such as buildings, highways, etc.) was carried out in different sections of the model at a scale of 1:2,000. Some attribute data was also added to the features during the digitizing operation. Oracle9i Spatial version used in this research has some visualization limits. Thus, accuracy evaluation and topological relationship control was carried out by the means of spatial SQL facilities of Oracle9i Spatial.

Results of IPOSS evaluation show the following abilities and advantages for this system:

- Ability to store and make structured data according to OGC standards simultaneously during the feature digitizing process from aerial and remote sensing images.
- Ability to detect and remove topological errors between different feature classes according to defined forbidden or unacceptable topological relations
- Ability to store and manage spatial and attribute data seamlessly in a spatial database at the time of spatial data production
- Ability to use stored data in GIS systems, especially in web based and on-line GIS, without additional preparation or editing process of the data.
- Ability to use Oracle Spatial facilities such as: spatial indexing, spatial analysis, recognition of topological relations, accuracy control, querying about stored spatial data etc. simultaneously during the feature digitizing process

Outcome of IPOSS is a set of fully structured spatial and attribute data which is stored in Oracle database based on OGC standards. Since topological and structural controls defined by the user have been carried out at the time of feature digitizing process, the set of data can be entered to GIS systems, especially in web based and on-line GISs, without additional preparation or editing process.

#### 4.5.2. An Applied Example

In this section, an application of IPOSS for digitizing of building feature is presented, as an applied example. In order to prepare IPOSS for the application; forbidden or unacceptable topological relations, between a building feature and other related feature classes, were recognized and expressed in the form of topological constraints. The following constraints show some of the forbidden constraints:

- Building Feature, Overlap, Building Feature
- Building Feature, Overlap, Road Feature
- Building Feature, Inside, Lake Feature
- Building Feature, Disjoin, Building Block Feature

These constraints were defined for IPOSS using its interface. This stage is known as the learning phase of the system.

**Figure 7.**
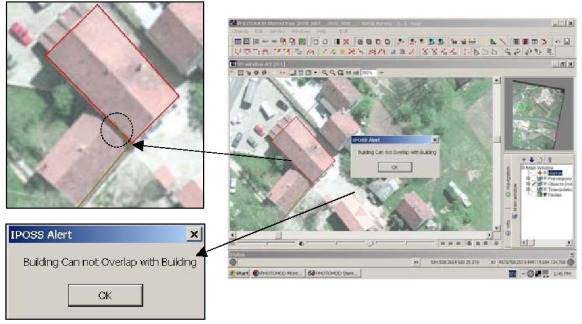
Sample operation of IPOSS System

After the learning stage, 3D model of an urban area was formed in PhotoMod using stereo aerial images at the scale of 1:8,000. Then, the digitizing process was carried out with emphasis on building features and other related feature classes. During this process whenever a forbidden constraint was occurred, IPOSS prevented error generation. Therefore, at the end of digitizing process the spatial data stored in database had no topological error. As shown in Figure 7, a building boundary has overlapped another building boundary and IPOSS has recognized this event as a forbidden topological relation and prevented error generation.

## 5. Conclusions and Recommendations

Direct integration of photogrammetric systems and SDBMSs removes the processes required for preparation of data for spatial information systems. So, this method saves time and cost of spatial data production. Spatial and attribute data management in a coupled approach is one of the important problems of GIS systems in using products of photogrammetric systems. Integration of the systems overcomes this problem by replacing digital maps with central spatial databases. This replacement also makes updating process more efficient.

In the IPOSS developed in this research, structuring of spatial data is carried out according to OGC standards and topological relations between features in the real world at the time of feature digitizing process. Therefore, by using IPOSS, acquired data can be entered to GIS systems, especially in web based and on-line GISs, without additional preparation or editing process. Also with synchronization of production, updating and sharing of spatial data, a great development in the field of on-line GIS can be achieved.

Some topological constraints do not have a fixed nature. These constraints are changing from one scale to another scale or from one application to another one. Therefore, a set of fixed constraints can not be used for detecting different kinds of topological errors. IPOSS enables experts to define topological constraints according to the scale and application by user interface of the system. So, IPOSS is a flexible and extensible system.

General gates for spatial data storing and exchanging such as Spatial Database Engine (SDE) allow users to apply different kinds of commercial DBMS to use, store and manage spatial data. Employing such a gate increases interoperability of the interface system.

Topological constraints have a critical role in automatic detection of topological errors. In other words; the knowledge required for error detection is provided by the constraints. Expert system architecture provides inference capability for the error detection section of the interfaces system. So, some required constraints which have not defined directly by users can be generated automatically form available constraints. Therefore, using architecture of expert systems in interface system designing is suggested for improving flexibility and automation level of the system.
